# Atorvastatin Rapidly Reduces Hepatitis B Viral Load in Combination with Tenofovir: A Prospective Clinical Trial

**DOI:** 10.1155/2022/3443813

**Published:** 2022-07-14

**Authors:** M. Gharehbeglou, S. Yazdani, K. White, M. R. Haeri, N. Masoumzadeh

**Affiliations:** ^1^Department of Anesthesiology and Critical Care Medicine, Faculty of Medicine, Shahid Beheshti University of Medical Sciences, Tehran, Iran; ^2^Department of Anesthesiology and Critical Care Medicine, Faculty of Medicine, Zahedan University of Medical Sciences, Zahedan, Iran; ^3^Department of Internal Medicine, School of Medicine, Qom Branch, Islamic Azad University, Qom, Iran; ^4^School of Human Sciences, London Metropolitan University, London, UK; ^5^Department of Clinical Biochemistry, Faculty of Medicine, Qom University of Medical Sciences, Qom, Iran; ^6^Department of Infectious Disease and Tropical Medicine, Imam Khomeini Hospital Complex, Tehran University of Medical Science, Tehran, Iran; ^7^Department of Infectious Disease and Tropical Medicine, School of Medicine, Zahedan University of Medical Science, Zahedan, Iran

## Abstract

**Method:**

In this single-blind clinical trial, 40 patients with active chronic hepatitis B were randomly allocated to treatment or control groups. The treatment group received the standard treatment for chronic HBV (300 mg tenofovir twice a day) along with 40 mg/day atorvastatin for 12 months, while the control group received a placebo once daily in addition to the standard tenofovir regimen. Serum aspartate aminotransferase (AST), alanine aminotransferase (ALT), and HBV DNA copy numbers were measured at the beginning of the treatment and 1, 3, 6, 9, 12 months later.

**Results:**

One month after starting the treatment, the HBV copy number in the atorvastatin + tenofovir-treated group was significantly lower, by 200×, compared with the control group. After three months of the treatment, there was no detectable HBV DNA in 50% of the atorvastatin + tenofovir-treated group compared with 30% in the control group. The half-life of plasma viral load was 2.03 and 3.32 months in the atorvastatin + tenofovir-treated and control groups, respectively. No adverse events due to taking atorvastatin were observed.

**Conclusions:**

The combination of atorvastatin with tenofovir increased antiviral activity and led to a faster recovery from viral infection. Therefore, this modality can be recommended as a safe combination therapy for chronic hepatitis B patients.

## 1. Introduction

Hepatitis B (HB) is the most common viral infection attacking the liver and can cause both acute and chronic diseases. In 2015, an estimated over 250 million people worldwide were living with chronic hepatitis B virus (HBV) infection. Most infected adults are able to successfully clear the hepatitis B virus and develop protective antibodies. However, some people are unable to get rid of the virus and will develop a chronic form of the infection [1]. Viral hepatitis caused 1.34 million deaths in 2015. Almost 720,000 deaths were due to cirrhosis and 470,000 deaths were due to primary liver cancer (hepatocellular carcinoma) [2].

Two established medications for the treatment of HBV are pegylated alpha-interferon (IFN) and an assortment of nucleoside and nucleotide analogs. IFN is effective, and there is no documented resistance of HBV to IFN, but the marked side-effects and expense are drawbacks [3]. In contrast, nucleotide analogs, such as tenofovir, inhibit HBV replication, are tolerated well, and are cheap, but the durability of the response to them is much poorer compared with IFN [3, 4]. A third therapeutic approach focuses on inhibiting the synthesis of cholesterol and related metabolites such as prenyl alcohol, through growing evidence of a strong relation between host cholesterol biosynthesis and the replication, assembly, or secretion of different viruses including HBV [5–9]. For example, lonafarnib, a candidate drug that was tested for treating hepatitis D, inhibits farnesyl transferase (an enzyme in the cholesterol biosynthesis pathway) and thus blocks prenylation [10].

Statins are a class of antihypercholesterolemic drugs that inhibit HMG-CoA reductase, a key, the rate-limiting enzyme in the cholesterol biosynthesis pathway [11]. Certain statins show antiviral activity against the hepatitis C virus (HCV) through inhibiting geranylgeranylation of proteins and hence HCV replication [12, 13]. Protein prenylation, which is blocked by statins, is necessary for HIV budding and spreading [14]. In the case of HBV, cholesterol has critical importance in the formation of the viral envelope and secretion [5]. Another study found that the presence of cholesterol in the viral envelope is necessary for the entry process of HBV [15]. Furthermore, 7-dehydrocholesterol, an immediate precursor of cholesterol is accumulated in HepG2 cells in a proportional manner to HBV replication. It was thereby suggested that HBV may selectively utilize 7-dehydrocholesterol for its replication [6]. These data clearly show a central role for intermediate metabolites of the cholesterol synthesis pathway in the HBV life cycle. *Aim*. In this clinical trial, we examined the combination of tenofovir and atorvastatin, a widely used antihypercholesterolemic drug, in comparison to tenofovir alone in the treatment of 40 patients infected with HBV who have not been treated with other HBV medications. The main goal of this study was to determine whether atorvastatin plus tenofovir can reduce HB viral load more effectively than tenofovir alone after 12 months of treatment.

## 2. Methods

### 2.1. Study Design

We selected 43 patients with active chronic hepatitis B that were absolute candidates for antiviral therapy, from April 2015 to April 2016. Inclusion criteria were a viral load of more than 100,000 copies/ml and elevated serum levels of liver enzymes alanine aminotransferase (ALT) and aspartate aminotransaminase (AST). Exclusion criteria included kidney failure, current treatment with immunosuppressive drugs, inborn immunodeficiency, autoimmune hepatitis, coinfection with HCV, HDV, or HIV, a history of taking cholesterol-lowering medication (statins) or antiviral drugs during the last 6 months, and liver disease including alcoholic and nonalcoholic steatohepatitis and cancer. All selected patients were HBe Ag negative, noncirrhotic based on sonographic pieces of evidence, and they did not have significant signs of fibrosis based on ALT and AST measurements (ALT/AST > 1). The size of the spleen and the portal vein diameter was normal. The platelet count was over 150,000 for all of the patients. Three patients were excluded based on the exclusion criteria. The number of participants required in this study was calculated according to statistical formulas. After selection, patients were randomly divided into two groups, test, and control, based on a table of random numbers and Epi Info software (Centers for Disease Control and Prevention, USA).

Enrolled patients from both sexes were between 20 and 76 years of age (median 43 years old) and read and signed informed consent forms before starting the treatment. The test group received the standard regimen for treatment of chronic HBV (300 mg tenofovir disoproxil fumarate twice a day) plus a conventional dose of 40 mg/day atorvastatin for 12 months. Higher doses of atorvastatin were not used, due to concerns of possible hepatotoxicity. The control group received the standard regimen for treatment of HBV (300 mg tenofovir twice a day, Hetero Healthcare) and a placebo for 12 months. Serum HBV viral load and liver enzymes were checked before starting the treatment and at the indicated intervals until the end of the study (0, 1st, 3rd, 6th, 9th, 12th month). Atorvastatin would have been withdrawn if patients showed any sign of elevated aminotransferase greater than 3 times their initial levels, but this was not necessary.

### 2.2. Blood Analysis

Realtime PCR using a Cobas Amplicor (Roche Diagnostics) was used to determine HBV DNA viral load, using a kit from Sacace Biotechnologies Srl (Como, Italy). ALT and AST levels were assayed using kits from Biosystems S. A. (Barcelona, Spain).

### 2.3. Statistical Analysis

Results were analyzed and compared by GraphPad Prism 8.2.1 software using the paired *t*-test and repeated measurement ANOVA and Kaplan–Meier survival analysis. The significance level was defined as *p* ≤ 0.05.

## 3. Results

40 patients completed the trial, 16 men (40%) and 24 women (60%), and the number of men and 20 women in both groups did not differ significantly (*p*=0.519). There were no significant differences between the atorvastatin + tenofovir-treated and control group in age (43.7 ± 14 vs. 43.9 ± 16 years, *p*=0.967), the mean baseline HBV DNA (1.671 ± 0.386 × 10^9^ vs. 1.606 ± 0.337 × 10^9^ copies/mL, *p*=0.267), and mean baseline AST (62 ± 13 vs. 64 ± 18 IU/L, *p*=0.753) and ALT (69 ± 13 vs. 72 ± 19 IU/L, *p*=0.527).

**Figure 1:**
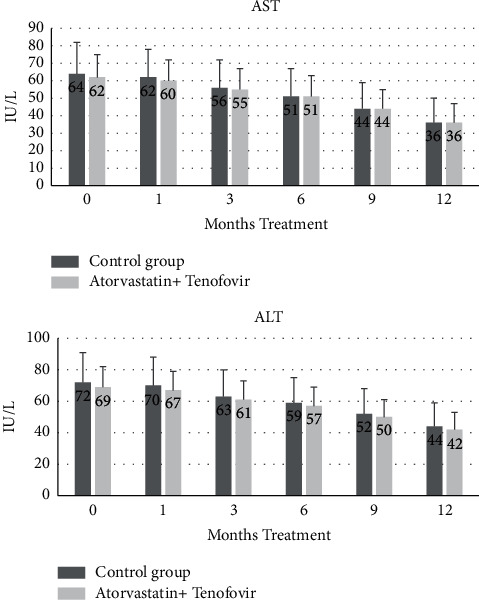
The effect of treatments on liver function. Serum levels of AST and ALT were measured in control patients treated with tenofovir (control) compared with patients treated with tenofovir and atorvastatin (atorvastatin + tenofovir). Data are the mean + SD of 20 patients and were analyzed by two-way ANOVA. At each time point, there is no significant difference between treatments, for either AST or ALT, but both treatments resulted in significantly decreased levels of enzymes compared with levels at the start of treatments (all *p* < 0.005).

There was no indication that patients receiving atorvastatin + tenofovir experienced enhanced liver damage compared with the control group (Figure 1). Indeed, in both groups, levels of serum ALT and AST declined steadily over the treatment course of 12 months, indicating a recovery of liver health, and at no time point was there a significant difference between the two groups.

**Figure 2:**
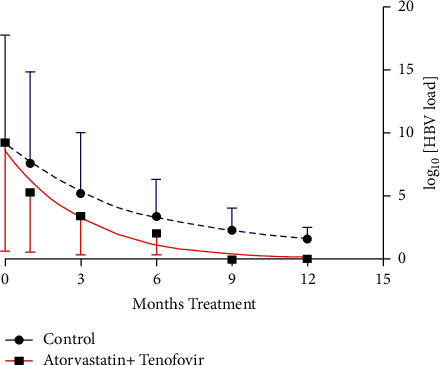
The effect of treatments on viral load. Data are mean + SD of viral load measured at indicated time points in the blood of 20 patients. A nonlinear curve was fitted to each set of data, from which the half-life of viral load in the plasma could be estimated.

After one month of treatment, HBV viral load in the atorvastatin + tenofovir-treated group decreased significantly more than the control group to 1.880 ± 0.543 × 10^5^ vs. 36.540 ± 17.968 × 10^6^ copies/ml, respectively (*p*=0.035), corresponding to a viral load decrease by 99.99% in the atorvastatin + tenofovir-treated group compared with 97.7% in the control group. However, a comparison of the mean HBV DNA copy number between the two groups did not show any significant differences in the 3rd, 6th, 9th, and 12th months of treatment (Figure 2).

After 3 months of treatment, 50% of the atorvastatin + tenofovir-treated patients and 30% of the control group patients had undetectable HBV DNA. After 6 months, 75% and 60%, after 9 months 100% and 90%, and after 12 months, 100% and 95% of atorvastatin + tenofovir-treated and control groups had undetectable HBV DNA, respectively. The half-lives of plasma viral load calculated from the curves in Figure 2 were 2.03 and 3.32 months for the atorvastatin + tenofovir-treated and control groups, respectively.

## 4. Discussion

Chronic HBV infection is a significant health problem worldwide and may cause serious complications such as cirrhotic liver failure and hepatocellular carcinoma (HCC) [16]. Statins are competitive inhibitors of 3-hydroxy-3-methylglutary-CoA (HMG-CoA) reductase, which is a rate-limiting enzyme involved in the production of cholesterol and several intermediary metabolites. Statins are among the most widely prescribed classes of medications, with more than 130 million prescriptions written in the United States in 2009 [17]. Statin use may reduce the risk of liver cancers in HBV-infected patients through inhibition of downstream products of cholesterol synthesis and HBV replication [13, 18].

In the present study, we investigated the effect of a combination of tenofovir and atorvastatin in comparison to tenofovir alone in the treatment of patients with hepatitis B. The conventional treatment for HBV-infected patients consists of 48 week (12 months) prescription of tenofovir. Following ethical obligations, we could not waive the conventional therapy for HBV patients. Therefore, in the intervention group, the patients received both tenofovir and atorvastatin from the beginning of the treatment for 48 weeks. Treatment with atorvastatin did not affect the gradual improvement of liver health during the study, as indicated by the serum markers AST and ALT (Figure 1). The mean AST and ALT levels were no different between atorvastatin and placebo at any time point, and by the end of the study, levels of both markers had decreased significantly. Furthermore, no severe adverse events were observed, and no rhabdomyolysis symptoms including musculoskeletal pain, concentrated urine, confusion, and vomiting were observed either. This indicates that atorvastatin can be given safely in combination with tenofovir.

The effect of the combination therapy on viral load was clear and marked. After one month, the viral load in patients receiving atorvastatin + tenofovir was 200× lower compared with the control group, and the improvement was sustained for the duration of the study (Figure 2). After nine months of treatment with atorvastatin + tenofovir, there was no detectable virus in patients, whereas in the control group treated with tenofovir only, detectable levels of virus were still present at the end of the 12-month study period. The rate of clearance of viral load was faster in the group treated with atorvastatin + tenofovir (viral load half-life 2.03 months) compared with the control group (viral load half-life 3.32 months). These data clearly demonstrate that atorvastatin could improve the effect of tenofovir in preventing virus replication.

The most probable explanation of this effect is due to the inhibition by atorvastatin of HMG-CoA reductase leading to depletion of cholesterol and its metabolites, resulting in decreased entry, replication, and release of HBV.

Cholesterol and/or its metabolic precursors, and lipid rafts, which are cholesterol-rich structures in membranes, play an important role in the entry, assembly, and budding of various viruses' life cycles [19–22]. For example, it was found that when human hepatoma HepG2 cells became infected with HBV, there was an enhanced expression of LDL receptors, to promote cellular uptake of cholesterol, and HMG-CoA reductase, to promote the cellular synthesis of cholesterol [23], which indicates that HBV has a marked need for cholesterol [23]. Moreover, simvastatin alone and in combination with nucleos(t)ide analogs, significantly suppressed HBV replication in vitro [3]. In another study, inhibition of cholesterol uptake in the intestine by ezetimibe was linked to a suppression of hepatitis B virus infection [24]. Consistent with these findings, depletion of cholesterol from cell membrane rafts leads to a sharp decrease in HBV release, and this effect is blocked after supplementation of cholesterol [25]. It has also been shown that HBV infection is dependent on the presence of cholesterol in the HBV envelope [15]. Furthermore, a close precursor of cholesterol, 7-dehydrocholesterol is incorporated into virions, and depletion of 7-dehydrocholesterol from cells reduced viral replication and virion secretion [6].

However, another possible mechanism for the anti-HBV effect of statins comes from the common transport system for statins and virus particles. Sodium taurocholate co-transporting polypeptide (NTCP) can be used as a receptor for HBV entry [26]. Virus entry through NTCP is blocked by Myrcludex B, which competes with virions for binding to the receptor [27, 28]. Surprisingly, some statins including atorvastatin are substrates for NTCP [29]. Statins may interact with NTCP and reduce HBV internalization. Interestingly, HDV uses the same way to enter hepatic cells [26, 27]. Therefore, there is a possibility that atorvastatin also blocks HDV entry and subsequent propagation. This suggests atorvastatin would be useful for treating not only HBV-infected but also HBV and HDV coinfected patients. However, further investigation is required to determine this interesting dual action of atorvastatin in HBV and HDV coinfected patients.

Atorvastatin and tenofovir help each other to reduce the viral load by two different mechanisms. Tenofovir inhibits virus replication and atorvastatin may act through one or all of those mentioned mechanisms to reduce the HBV replication. The combined effect of them leads to a more effective recovery of patients with proven HBV, which was seen in our study as one of the first to demonstrate the safe, anti-HBV activity of a statin.

## 5. Conclusion

Our results show that a combination of atorvastatin with tenofovir reduces HB viral load faster than tenofovir alone. Atorvastatin is a cheap, widely available, and safe drug that can be used at conventional doses in combination therapy with an anti-HBV drug in current use, tenofovir, to improve the treatment protocol for HBV-infected patients, for eradicating HBV more rapidly than tenofovir alone.

## Data Availability

The data used to support the findings of this study are available upon request from the corresponding author.

## Additional Points

*Key Points*. (i) Statins are now believed to interfere with some virus replication processes such as budding from infected cells. (ii) Atorvastatin, with the help of tenofovir, improves antiviral activity and led to a faster recovery from the hepatitis B virus. (iii) Atorvastatin could reduce HBV copy number by 200 times compared with the control group. (iv) Atorvastatin can be recommended as a safe adjuvant for treating chronic hepatitis B patients.
